# Characterization of a novel *Leishmania* antigen containing a repetitive domain and its potential use as a prophylactic and therapeutic vaccine

**DOI:** 10.1128/msphere.00097-25

**Published:** 2025-04-22

**Authors:** Bianca de Oliveira, Wanessa M. Goes, Frederico C. Nascimento, Juliana B. T. Carnielli, Eden R. Ferreira, Alex Fiorini de Carvalho, Pablo Victor Mendes dos Reis, Milton Pereira, Tiago Queiroga Nery Ricotta, Liliane Martins dos Santos, Renan Pedra de Souza, Diego Esteban Cargnelutti, Jeremy C. Mottram, Santuza R. Teixeira, Ana Paula Fernandes, Ricardo T. Gazzinelli

**Affiliations:** 1 Centro de Tecnologia de Vacinas (CTVacinas), Universidade Federal de Minas Gerais, Belo Horizonte, Minas Gerais, Brazil; 2 Instituto René Rachou, Fundação Osvaldo Cruz—Minas, Belo Horizonte, Minas Gerais, Brazil; 3 Faculdade de Farmácia, Universidade Federal de Minas Gerais, Belo Horizonte, Minas Gerais, Brazil; 4 York Biomedical Research Institute, Department of Biology, University of York, York, United Kingdom; 5 Instituto de Medicina y Biología Experimental de Cuyo (IMBECU), Consejo Nacional de Investigaciones Científicas y Técnicas (CONICET), Universidad Nacional de Cuyo (UNCuyo), Mendoza, Argentina; Virginia-Maryland College of Veterinary Medicine, Blacksburg, Virginia, USA

**Keywords:** vaccines, leishmaniasis, recombinant protein production, adjuvants, immune response, protein localization

## Abstract

Human visceral leishmaniasis (HVL) is the second most lethal tropical parasitic disease. Currently, no prophylactic or therapeutic vaccines exist for HVL. Thus, the development of an efficacious vaccine is still needed. We previously performed an immunoproteomics analysis on *Leishmania amazonensis* parasite extracts to identify immunodominant antigens recognized by the sera of vaccinated and protected mice. Among the identified antigens, we discovered a novel, previously unstudied repetitive protein, initially annotated in *Leishmania* genomes as a kinetoplast-associated protein-like protein from *Leishmania infantum* (LinKAP), containing conserved domains (trichohyalin-plectin-homology [TPH] and TolA) that are associated with other mitochondrial proteins. LinKAP sequences are conserved across trypanosomatids, including *Endotrypanum, Leishmania,* and *Trypanosoma* species. Using differential centrifugation of *Leishmania* subcellular structures, we showed that LinKAP was enriched in fractions colocalizing with other mitochondrial proteins. mNeonGreen labeling at the endogenous locus using CRISPR-Cas9 and confocal microscopy confirmed that LinKAP is a mitochondrial-associated protein in *Leishmania* but not specifically colocalized with kDNA. We cloned and expressed a truncated version of LinKAP (rLinKAP), containing part (15) of the several LinKAP amino acid repeats, demonstrating over 85% homology across *L. infantum, L. amazonensis, L. braziliensis,* and *L. mexicana* species. An adjuvanted formulation of LinKAP with Poly ICLC, a polyinosinic-polycytidylic acid (Poly I:C) stabilized with carboxymethylcellulose and polylysine, was used to vaccinate mice and hamsters as a prophylactic vaccine for visceral leishmaniasis. Animals immunized with rLinKAP showed a potent cellular and humoral response and a significant decrease in tissue parasitism when challenged with *L. infantum*. We also tested rLinKAP as a therapeutic vaccine in mice. Following therapeutic vaccination, antibody responses were enhanced, and cellular responses became apparent. Our treatment protocol inhibited splenic parasite burden by 75% in treated mice. In conclusion, our antigen discovery strategy and the observed protective effect highlight rLinKAP as a promising vaccine candidate for leishmaniasis.

**IMPORTANCE:**

A previous reverse vaccinology study identified kinetoplast-associated protein-like protein from *Leishmania infantum* (LinKAP) as a potential new vaccine target, as this protein was recognized by the sera of protected mice in extracts of *Leishmania* promastigotes. Interestingly, LinKAP is a repetitive protein containing trichohyalin-plectin-homology (TPH) and TolA domains and was initially annotated as a kinetoplast-associated protein. We further characterized LinKAP as a mitochondrial-associated protein highly conserved among trypanosomatids. We also validated LinKAP as a promising vaccine antigen by using a truncated version of LinKAP (rLinKAP) as both a prophylactic and therapeutic vaccine, adjuvanted with Poly ICLC, to immunize animals against visceral leishmaniasis (VL). This disease, caused by the *Leishmania* parasite, affects several populations globally and still lacks highly effective vaccines. Identifying LinKAP and its preliminary characterization also provides new perspectives for studying its role in the parasite's biology.

## INTRODUCTION

Human leishmaniasis (HL), a cluster of parasitic diseases intricately linked to impoverished regions, threatens over one billion people globally (1). There are approximately 53 different species of *Leishmania* spp., 20 of which have been found to infect humans (2). The most severe form of HL, visceral leishmaniasis (VL), caused by *Leishmania donovani* and *L. infantum* species and less frequently by *L. amazonensis* may present an alarming fatality rate of up to 95% when left untreated. Annually, 50,000 to 90,000 new VL cases are reported, predominantly in East Africa, India, and Brazil (1, 3, 4). Currently, VL treatment and control rely on chemotherapy and diagnostics. However, the complexity of immune responses, parasite variability, and host interactions with various *Leishmania* species significantly impact the efficacy of these tools (5, 6).

Most VL patients develop long-term protective immunity after recovering from infection, suggesting the potential for an effective prophylactic strategy (6). Despite the availability of vaccines for canine VL, the absence of a human VL vaccine poses a significant challenge to disease control (6). In this context, therapeutic vaccines hold considerable promise. Since drug efficacy depends on the synergistic activity of immune responses, the potential for pharmacological interventions is limited. In addition, the emergence of drug resistance has become a critical concern in various regions (7–9).

There is significant consensus that an effective VL vaccine should trigger a strong and lasting Th1 cellular immune response (10). Among the cytokines T cells produce, interferon gamma (IFN-γ) plays a vital role in activating macrophages, eliminating *Leishmania* parasites (11). In contrast, a Th2 response, characterized by the production of the anti-inflammatory cytokine interleukin-10 (IL-10), deactivates infected macrophages, promoting disease progression. The balance between IFN-γ and IL-10 production is crucial, as it influences the levels of IgG2a and IgG1 isotype antibodies, which serve as biomarkers of host resistance and susceptibility to infection (11, 12). Therefore, an effective immune response against VL requires the induction of a Th1 cell-driven cascade, characterized by elevated IFN-γ, alongside other pro-inflammatory cytokines and the production of anti-*Leishmania* IgG2a antibodies.

Recombinant protein-based vaccines offer several advantages, including enhanced stability, cost-effectiveness, and standardized production protocols (13, 14). Recombinant protein vaccine formulations, encompassing known and hypothetical *Leishmania* proteins, have been evaluated in murine and canine models, revealing key features of robust immune responses and demonstrating protection against *L. infantum* infection (15–23). In this context, identifying new antigen candidates for effective VL vaccine development may further improve vaccine formulations.

Immunoproteomics has proven to be an effective strategy for antigen discovery targeting pathogenic agents (24–27). Recently, we employed this approach to identify novel antigen candidates (10). First, we immunized mice with total *L. amazonensis* antigens (TLAs) combined with Poly ICLC and Montanide ISA 763. This protocol reduced parasite burden and lesion size in vaccinated animals challenged with *L. amazonensis*. Subsequently, immunoblot-proteomic analysis was conducted, identifying four immunodominant proteins as promising candidates for vaccine development, some of which had never been assessed for this specific purpose (24).

One of these proteins, the kinetoplast-associated protein-like protein from *Leishmania infantum* (LinKAP), contains multiple repetitive amino acid units and remains largely uncharacterized. Here, we characterized the molecular and biochemical properties of this protein. In both prophylactic and therapeutic protocols, immunization with recombinant LinKAP (rLinKAP), combined with Poly ICLC, conferred immune-mediated resistance to *L. infantum* infection. Our findings may contribute to the collective effort in developing vaccines for leishmaniasis, a global health threat.

## RESULTS

### LinKAP is a protein containing amino acid repeats and is conserved in several *Leishmania* species

The LinKAP protein is encoded by a single copy gene, located on chromosome 27 of *L. infantum* (*TritrypDB* gene ID: LINF_270007500), which is originally annotated as a kinetoplast-associated protein-like with a theoretical molecular weight of 72 kDa. This gene is exclusively present in trypanosomatids, and it has been described, so far, only in species of the genera *Leishmania, Trypanosoma,* and *Endotrypanum*, which are digenetic parasites (28).

**Fig 1:**
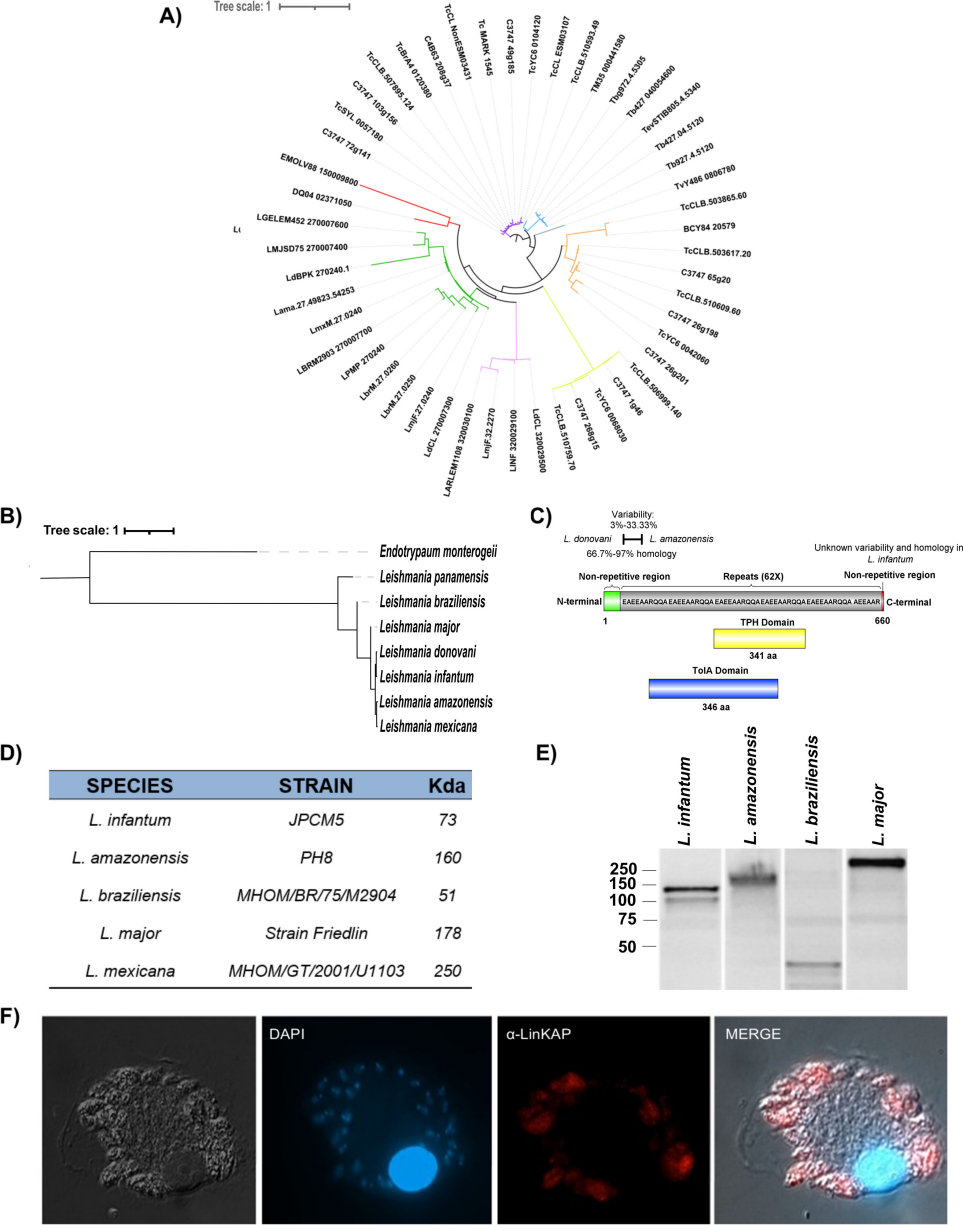
Conservation and expression of the LinKAP protein in pathogenic trypanosomatids. (**A**) Maximum-likelihood phylogenetic tree of 49 LinKAP sequences, constructed using the LG + I substitution model for protein alignment. *Leishmania*-specific clades are shown in green and pink, while *Trypanosoma*-specific clades are represented in orange, dark blue, light blue, purple, and yellow. The red color denotes a mixed clade consisting of *Endotrypanum monterogeii* and *Trypanosoma grayi*. Different colors within the same genus indicate distinct evolutionary origins and potentially different functional classes of the LinKAP protein. (**B**) Consensus phylogenetic tree of LinKAP proteins within the *Leishmania* genus. The *Endotrypanum monterogeii* protein sequence was used as an outgroup. (**C**) Schematic representation of the LinKAP protein from *L. infantum* strain JPCM5. The variability and degree of homology with other *Leishmania* species are also depicted in the upper section. (**D**) Predicted molecular weights of LinKAP across various *Leishmania* species, highlighting species-specific variations. (**E**) Western blot analysis of LinKAP expression in multiple *Leishmania* species. Total protein extracts were prepared from logarithmic-phase cultures and probed with anti-LinKAP antibodies, revealing distinct expression patterns among species. (**F**) Immunofluorescence analysis of *L. mexicana*-infected macrophages. Macrophages were infected with *L. mexicana* promastigotes and incubated for 48 h to allow differentiation into amastigote forms. Cells were then fixed with paraformaldehyde and stained with an anti-LinKAP antibody, highlighting LinKAP expression within intracellular amastigotes. Parasite nuclei and kinetoplasts were counterstained with DAPI (blue). Images were acquired using a 100× objective, confirming the presence of LinKAP in the amastigote stage inside macrophages.

LinKAP contains repetitive amino acid units—comprising approximately 60% of the protein—that are highly conserved among *Leishmania*, *Endotrypanum*, and *Trypanosoma*. A search for orthologous genes in the *TritrypDB* database identified 49 amino acid sequences that share similarities with the LinKAP protein. A phylogenetic tree, constructed using PhyML 3.0.1 revealed the presence of genus-specific gene sequences, distinctly forming separate clades that differentiate *Leishmania* from *Trypanosoma* (Fig. 1A).

The *Endotrypanum* sequences were used as an outgroup to root the tree (29). As expected, sequences of species belonging to *Leishmania* and *Viannia* subgenera were clustered into distinct clades (30). LinKAP sequences from the subgenus *Leishmania* displayed a high degree of evolutionary conservation, forming a single cluster (Fig. 1B). Conservation among species within the same complex remained evident, as further highlighted by the alignment of LinKAP sequences from the main causative species of visceral leishmaniasis (VL) and cutaneous leishmaniasis (CL) (Fig. S1). These data indicate that LinKAP has been preserved under strong selective pressure in the genomes of *Leishmania spp*. and *Trypanosoma spp*. However, a better understanding of the evolutionary history of LinKAP proteins requires improvements in the sequencing and annotation of these and other trypanosomatids genomes.

The analysis of the complete 660-amino-acid sequence of the LinKAP protein revealed a disordered region with homology to a trichohyalin-plectin-homology (TPH) domain and a TolA protein (Fig. 1C). These domains are associated with mitochondrial motility in eukaryotes and the uptake of colicins and filamentous DNA in bacteria, respectively (31). Significant statistical results for the TPH domain, with a hit interval identified between amino acids 260 and 475, resulting in a total length of 346 amino acids. The PSSM ID for this domain is 274303, which achieved a bit score of 78.35 and an E-value of 1.94e−15, indicating a high level of statistical significance for the identified homology. The coverage of this homology within the complete LinKAP sequence is approximately 32.73%, suggesting that around one-third of the total sequence aligns with or is related to the TPH domain, demonstrating substantial conservation across related proteins.

The analysis of the TolA domain within the LinKAP protein also revealed a significant hit, with parameters consistent with those found for the TPH domain. Specifically, the interval for the TolA domain spans amino acids 260 to 475, corroborating its classification as a multi-domain protein with a conserved length of 346 amino acids. The bit score and E-value are identical to those of the TPH domain, reinforcing the high statistical significance of this alignment. The coverage of the TolA domain within the LinKAP sequence mirrors that of the TPH domain at approximately 32.73%. This substantial coverage emphasizes the evolutionary conservation of the TolA domain across related proteins, further supporting the functional relevance of LinKAP in the context of *Leishmania* species. However, further analyses are needed to elucidate the functional implications of these domains within the LinKAP protein.

Western blot analysis was performed using a polyclonal antibody produced in rabbits immunized with a fragment of the LinKAP protein (Fig. 3D) to evaluate its expression in promastigotes of different *Leishmania* species. The results shown in Fig. 1E demonstrate that LinKAP is expressed in this parasite stage by all the *Leishmania* species analyzed. The observed protein molecular weights generally matched those predicted by the annotated sequences, except for the *L. infantum* and *L. braziliensis* extracts in Fig. 1D. These discrepancies may be attributed to the quality of the available sequence data. Inaccuracies can arise in regions containing repetitive sequences, often leading to a misestimation of the number of repeats. Furthermore, repetitive regions are more prone to rearrangements, and repetitive proteins may exhibit variations in repeat numbers among different species or even within the same parasite family (32–36). Additionally, bioinformatic analysis of *Leishmania* sequences using the NetNGlyc 1.0 program (37) identified potential glycosylation sites in LinKAP. Such post-translational modifications may also affect protein migration in SDS-PAGE, introducing deviations from the expected molecular weight (38).

Through immunofluorescence of macrophages infected with *L. mexicana*, we observed that LinKAP is also expressed in the amastigote form of the parasite (Fig. 1F). To validate the specificity of the observed signal, a negative control without the primary antibody was used (Fig. S2). This finding corroborates the hypothesis that LinKAP plays a relevant role in the parasite’s life cycle, including the expression in the parasite intracellular stage, which may have functional implications for host-parasite interactions.

### LinKAP is a most likely mitochondrial-associated protein from *Leishmania*

**Fig 2:**
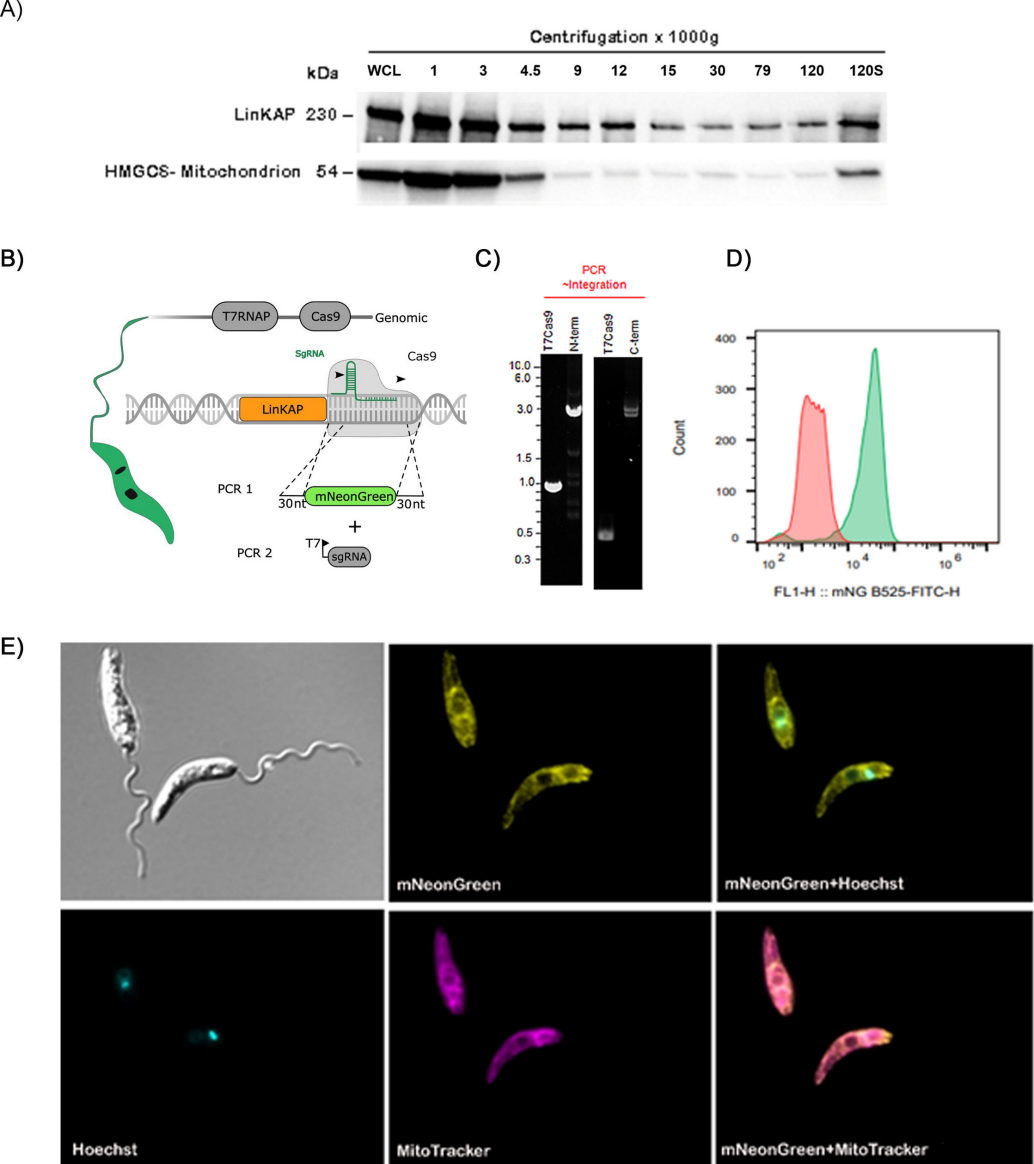
LinKAP is identified as a mitochondrial protein in *Leishmania*. (**A**) Western blot analysis of *L. mexicana* lysate fractions following differential centrifugation. Each fraction contains 10 µg of total protein, and the blot was probed with an anti-rLinKAP antibody, revealing a protein profile consistent with mitochondrial localization. WCL represents the whole cell lysate, while 120S indicates the final supernatant fraction. (**B**) Schematic representation of the C-terminal tagging strategy, illustrating the integration of a fluorescent tag and an antibiotic resistance gene. Transfection with sgRNA (PCR1) directs a double-stranded break upstream of the coding sequence (CDS), while the repair template (PCR2) includes 30-nucleotide homology arms to enable homologous recombination. (**C**) PCR verification of mNeonGreen (mNG) tag integration at the N- and C-termini, establishing the 3×Myc::mNG::LinKAP cell lines. (**D**) The flow cytometry histogram shows mNeonGreen fluorescence. The parental *L. mexicana* Cas9 T7 cell line (red) serves as a negative control, while the 3×Myc::mNG::LinKAP cell line with C-terminal tagging (green) confirms successful tagging and LinKAP expression in live cells. (**E**) Fluorescence microscopy of live promastigotes from the 3×Myc::mNG::LinKAP *L. mexicana* cell line, tagged with MitoTracker for mitochondria and stained with Hoechst 33342 for nuclear and kinetoplast DNA. This imaging shows LinKAP localization throughout the mitochondrial region.

The subcellular localization of the LinKAP protein was assessed in *L. mexicana* organelles by applying nitrogen cavitation to better preserve intracellular compartments, followed by differential centrifugation (39). To identify the subcellular localization of LinKAP, its distribution profile was compared with known organelle markers, including proteins from the nucleus, mitochondria, cytoskeleton, glycosomes, cytosol, and endoplasmic reticulum. Although LinKAP was detected in all fractions, it was enriched in low-speed pellet fractions, which correspond to mitochondria, during the initial centrifugation steps—a distribution pattern also observed with the mitochondrial marker HMGCS. (Fig. 2A). Densitometric analysis of the Western blots for each fraction further confirmed these observations, enabling the identification and comparison of LinKAP distribution across organelle-specific fractions (Fig. S3).

To further investigate LinKAP localization, Cas9-mediated genome engineering was used (Fig. 2B) to endogenously tag both alleles of LinKAP at either the N- or C-terminus with 3×Myc fused to mNeonGreen (mNG), generating the 3×Myc::mNG::LinKAP and LinKAP::mNG::3×Myc cell lines (Fig. 2C). Bioinformatic analysis identified no mitochondrial import signal in the N-terminal region of LinKAP, suggestive that N-terminal tagging would not interfere with its mitochondrial localization.

**Fig 3:**
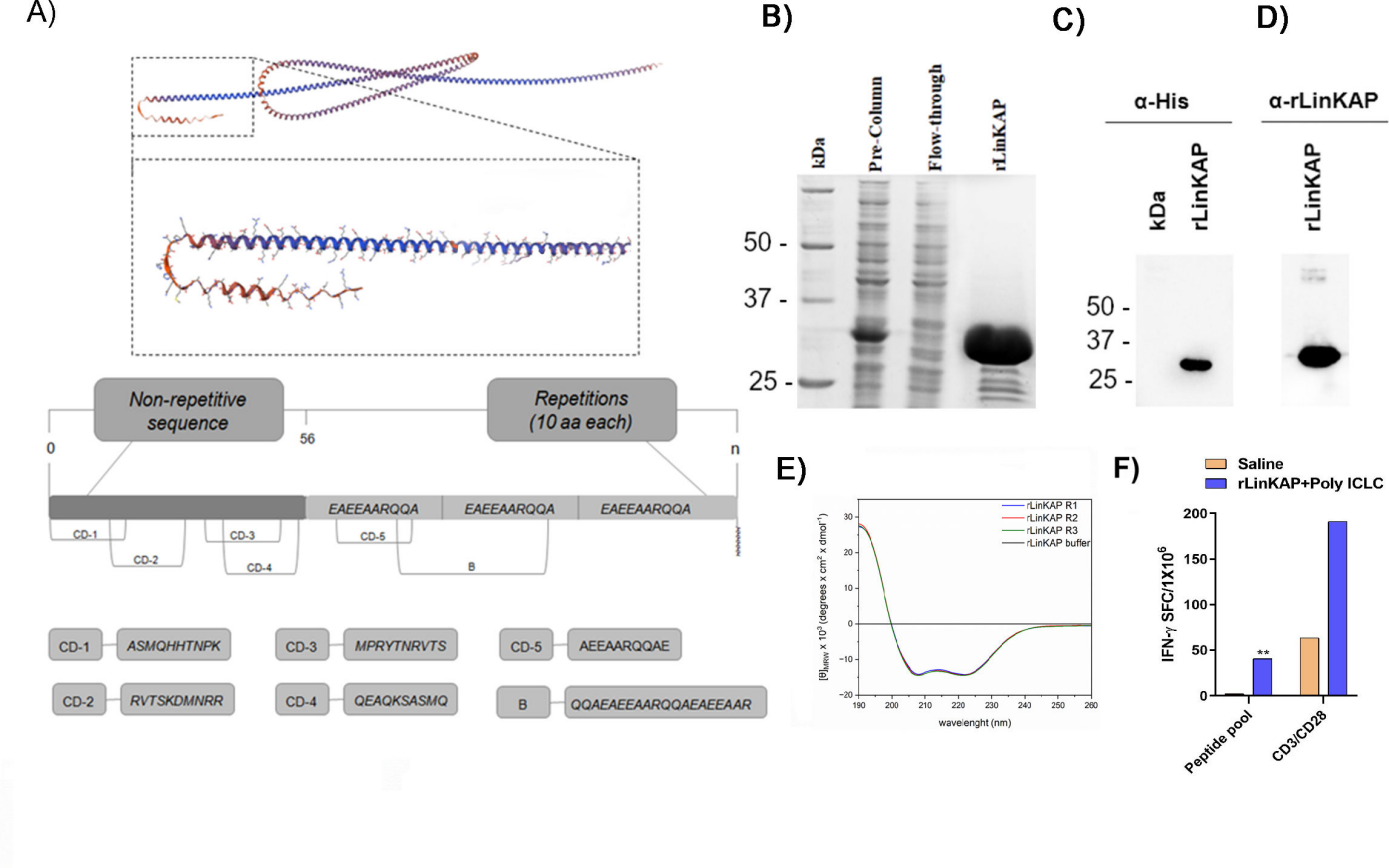
The recombinant protein LinKAP (rLinKAP). (**A**) Structural model of the LinKAP protein with highlighted regions for predicted T-cell and B-cell epitopes specific to *Leishmania infantum*. (**B**) SDS-PAGE analysis with Coomassie Blue staining showed rLinKAP protein elution fractions, confirming successful expression and purification. (**C**) Western blot of purified rLinKAP using an anti-histidine antibody, revealing a prominent band at the expected molecular weight for the recombinant protein. (**D**) Western blot analysis demonstrating the reactivity of the rabbit polyclonal antibody with rLinKAP, explicitly marking the expected molecular weight band. (**E**) Determination of the secondary structure of rLinKAP. The Jasco 1500 spectropolarimeter was used to read the molar ellipticity spectra. After data acquisition, the spectra were processed by the Bestsel internet server. (**F**) ELISpot assay results show the mean IFN-γ responses in splenocytes from immunized and non-immunized BALB/c mice stimulated with peptide pools CD1, CD2, CD3, CD4, and CD5. Data are expressed as spot-forming cells (SFC) per 1 × 10^6^ cells after subtracting background values (RPMI-only wells). Bars represent the mean. Statistical significance is indicated as **P < 0.01.

Both constructs exhibited similar expression and localization patterns, with the C-terminally tagged construct selected for imaging and analysis. Flow cytometry of the LinKAP::3×Myc::mNG cell line demonstrated LinKAP expression in approximately 90% of the promastigote cells, with the parental *L. mexicana* Cas9 T7 strain used as a negative control (Fig. 2D). Additionally, live-cell fluorescence imaging of LinKAP::3×Myc::mNG labeled with MitoTracker revealed that LinKAP is primarily located throughout the mitochondrial region (Fig. 2E). Overlap between LinKAP and Hoechst channels indicated that LinKAP does not specifically co-localize with the kinetoplast but is instead distributed throughout the mitochondria. A similar localization pattern was observed for LinKAP tagged at its N-terminus (data not shown). This finding was further confirmed by confocal microscopy analysis using a rabbit-produced polyclonal anti-LinKAP antibody (Fig. 3D). The observed signals strongly suggest that LinKAP associates with the mitochondria in both *L. infantum* and *L. amazonensis*, as shown in Video S1 and Video S2 (https://figshare.com/s/a8dbca7518153e3e9219).

### Recombinant LinKAP as a vaccine candidate

A recombinant version of the protein, containing a truncated portion of the LinKAP sequence, was designed based on epitope prediction as a candidate antigen for visceral leishmaniasis (VL). Epitope mapping was performed using the Immune Epitope Database and Analysis Resource (IEDB) platform for class I analysis and NetMHCII for class II, encompassing epitopes for both B and T cells. The selected region includes conserved epitopes found in various *Leishmania* species.

Subsequently, *in silico* analysis revealed that LinKAP contains multiple conserved epitopes, leading to the identification of an immunogenic peptide pool (Fig. 3A). The recombinant protein LinKAP (rLinKAP) comprises 54 amino acids in the N-terminal region, incorporating epitopes for both B and T cells, and contains 15 repeats of 10 amino acids derived from LinKAP. The predicted molecular mass of rLinKAP is approximately 23 kDa, with an isoelectric point of 4.77. Accordingly, the construct was expressed in *Escherichia coli* and purified for subsequent validation.

Polyacrylamide gels and immunoblotting confirmed the presence of highly purified rLinKAP protein expressed in *Escherichia coli*, with a molecular mass slightly different from the theoretical value (Fig. 3B and C). However, MALDI-TOF mass spectrometry confirmed a mass consistent with the theoretical value (Table S1). As previously mentioned, this discrepancy is commonly observed in repetitive proteins from *Leishmania* (32–36). Additionally, LPS quantification confirmed minimal endotoxin contamination, with levels below the recommended limit for vaccines (25 U/10 µg dose), thereby reinforcing the protein’s suitability for immunization.

Immunoblotting analysis using a rabbit-produced polyclonal antibody against rLinKAP revealed a clear band corresponding to the purified rLinKAP (Fig. 3D). Immunoblotting of total protein extracts from promastigotes of different *Leishmania* species confirmed that this polyclonal antibody detects LinKAP in complex samples (Fig. 1E).

Secondary structure analysis was performed to gain insight into the structural properties of rLinKAP. The analysis revealed that rLinKAP predominantly adopts flexible and disordered conformations, classified as “other” structures (41.43% ± 0.45%), followed by alpha-helix (25.73% ± 0.65%), antiparallel beta-sheet (12.83% ± 0.82%), turns (11.80% ± 0.14%), and parallel beta-sheet (8.20% ± 0.54%) (Fig. 3F; Table 1). These findings suggest that rLinKAP has a highly flexible conformation.

**TABLE 1:** Deconvolution of the rLinKAP protein^*a*^

Secondary structure	Percentage			Average	Standard deviation
α-Helix	24.90	26.50	25.80	25.73	0.65
β-Sheet parallel	7.60	8.10	8.90	8.20	0.54
β-Sheet antiparallel	13.90	12.70	11.90	12.83	0.82
Turn	11.90	11.90	11.60	11.80	0.14
Others	41.70	40.80	41.80	41.43	0.45

^*a*^ After data acquisition, the spectra were processed by the Bestsel web server.

To assess the immunostimulatory potential of rLinKAP, we evaluated by ELISpot the IFN-γ production in splenocytes stimulated with the peptide pool. The analysis demonstrated a significant increase in IFN-γ-secreting cells, confirming a strong T-cell response (Fig. 3G). As IFN-γ is a key cytokine associated with macrophage activation and Th1 immune responses, these results suggest that rLinKAP elicits a robust cell-mediated immune response against *Leishmania*. Together, these findings support its potential as a vaccine candidate for VL.

### Immunoprotective responses of animals vaccinated with rLinKAP

**Fig 4:**
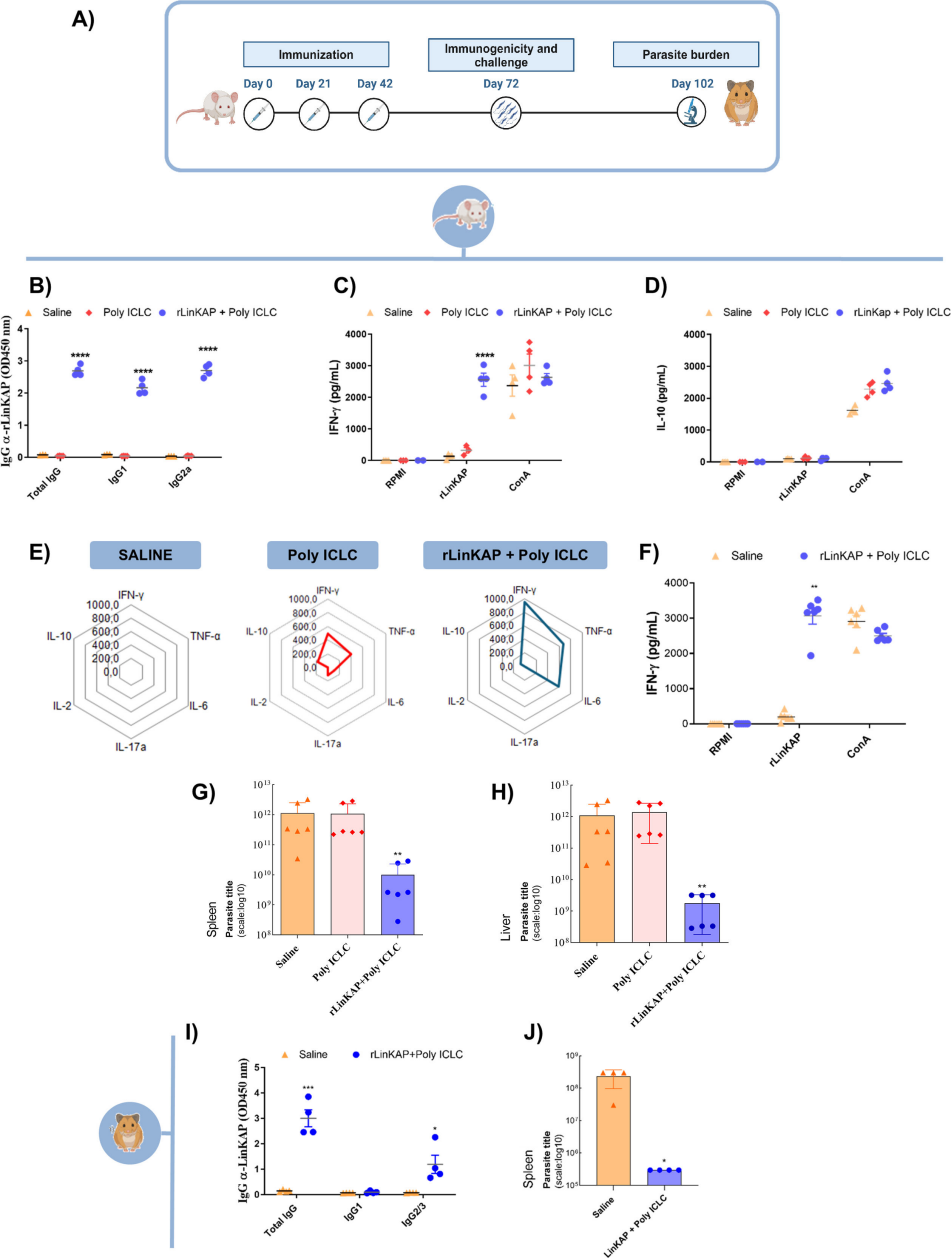
rLinKAP as a prophylactic vaccine. (**A**) An immunotherapy protocol was used to analyze the immune response and protect against *L. infantum* infection. (**B**) Evaluation of humoral immune response was conducted 30 days after immunization. Serum samples from animals (dilution 1:500) were used to detect total IgG, IgG1, and IgG2a antibodies specific to rLinKAP. (**C**) Cytokine production in response to stimulation with rLinKAP proteins in immunized animals. Splenocytes were cultured, and supernatants were used for IFN-γ and (**D**) IL-10 analysis by ELISA 48 h post-stimulation. (**E**) The frequency of cytokine production for the LinKAP protein group was depicted on a radar chart created with the mean of each cytokine, with error bars representing the standard deviation for each measurement, as assessed by cytometric bead array (CBA). (**F**) Splenocytes were cultured 82 days after the third dose of rLinKAP, and supernatants were used for IFN-γ analysis 48 h after stimulation with recombinant protein. (**G, H**) Parasite titer analysis in the spleen (**G**) and liver (**H**) 30 days post-challenge with *L. infantum* infection in BALB/c mice immunized with the recombinant antigen. Data are presented as parasite titer (log10 scale) per organ homogenate. (**I**) IgG antibodies and antigen-specific subclasses were measured in the serum of vaccinated and non-vaccinated hamsters 30 days after the last vaccination dose with rLinKAP antigen. (**J**) Spleen tissue analysis 30 days post-challenge with *L. infantum* infection in hamsters immunized with the recombinant antigen, quantified by limiting dilution. Data are presented as parasite titer (log10 scale) per spleen homogenate. Using the log10 scale in panels G, H, and J ensures consistency across parasite titer measurements. Statistical significance is indicated as *P < 0.05, **P < 0.01, and ***P < 0.001. Dots represent individual values for each animal.

Next, we assessed the immunogenicity of rLinKAP and its potential to confer protection against *L. infantum* infection. Mice received three subcutaneous doses of rLinKAP protein at 21-day intervals, using Poly ICLC (a synthetic adjuvant containing carboxymethylcellulose and polylysine stabilizers) as an adjuvant (Fig. 4A).

As shown in Fig. 4B, vaccination with rLinKAP + Poly ICLC elicited strong anti-rLinKAP antibody responses, with notably high IgG2a levels. Given the critical role of cellular immunity in combating *Leishmania* infection, IFN-γ levels were measured in splenocyte cultures from immunized mice stimulated with rLinKAP for 48 h. Splenocytes from rLinKAP + Poly ICLC-immunized mice exhibited significantly higher IFN-γ production than controls (Fig. 4C). Additionally, IL-10 levels and other cytokines associated with parasite persistence were assessed, as their modulation is linked to disease progression. Following rLinKAP stimulation, IL-10 levels remained low across all immunized groups (Fig. 4D).

The cytokine production profile, represented in a radar plot, showed that the rLinKAP-containing vaccine elicited a pro-inflammatory response, marked by high TNF-α and IFN-γ levels, with low IL-10 and IL-4 levels, suggesting a Th1-biased immune response (Fig. 4E). To evaluate the long-term durability of the Th1 response, IFN-γ levels were measured 90 days after the second and third doses, confirming a sustained Th1 profile, essential for effective leishmaniasis protection (Fig. 4F).

Following the *L. infantum* challenge, mice immunized with rLinKAP + Poly ICLC showed a significant reduction in parasite burden in the liver and spleen, as measured by limiting dilution assays, compared with saline and adjuvant-only controls. (Fig. 4G and H).

The formulation was also tested in hamsters, an established model for progressive visceral leishmaniasis. Hamsters immunized with rLinKAP + Poly ICLC displayed high serum IgG levels, specifically IgG2/3, without detectable IgG1 production (Fig. 4I). Following *L. infantum* challenge, vaccinated hamsters maintained stable body weight and survived, with significantly lower splenic parasite burdens compared with non-vaccinated controls (Fig. 4J). These results underscore rLinKAP as a promising candidate for a vaccine against *Leishmania* infection.

### Immunotherapeutic efficacy of rLinKAP in treated animals

**Fig 5:**
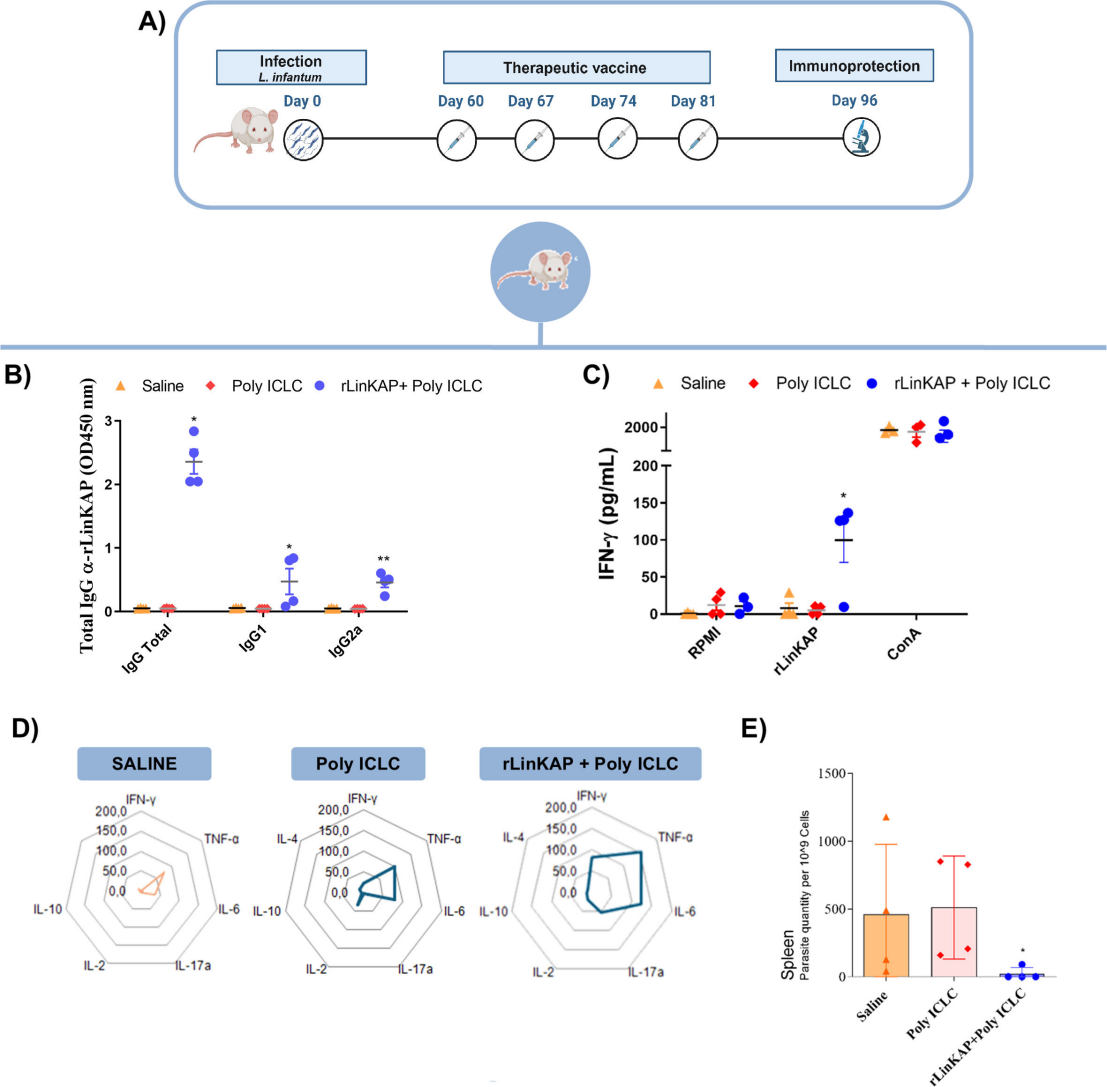
rLinKAP as a therapeutic vaccine. (**A**) Schematic representation of the therapeutic protocol applied to assess immune response and treatment efficacy. (**B**) Assessment of humoral immune response 15 days post-treatment, with total IgG, IgG1, and IgG2a antibodies specific to rLinKAP measured in serum samples (1:5,000 dilution). (**C**) IFN-γ production in splenocytes from infected and treated animals stimulated with rLinKAP protein was analyzed by ELISA after 48 h of culture. (**D**) The radar graph displays the mean cytokine production frequency for each cytokine in the rLinKAP-treated group, measured by the cytometric beam array (CBA). (**E**) Parasite load in the spleen of BALB/c mice 15 days post-infection and treatment with rLinKAP. Statistical significance is represented as *P < 0.05, **P < 0.01, and ***P < 0.001. Dots represent individual values for each animal.

The immunotherapeutic potential of the rLinKAP + Poly ICLC formulation against visceral leishmaniasis (VL) was evaluated by administering repeated doses to *L. infantum*-infected BALB/c mice (Fig. 5A). Fifteen days after the final dose, humoral responses were assessed, revealing significantly elevated titers of anti-rLinKAP IgG, IgG1, and IgG2a in treated mice compared with control groups (Fig. 5B).

IFN-γ production was quantified in rLinKAP-stimulated splenocyte supernatants, with treated mice displaying significantly higher levels than controls (Fig. 5C). To determine whether therapeutic vaccination induced a cytokine profile similar to that observed in prophylactic vaccination, we analyzed cytokine production patterns in infected and treated groups, given that a robust Th1 response provides a rational basis for effective therapeutic intervention (40).

The immune response analysis revealed distinct cytokine production patterns. The saline-treated control group exhibited low TNF-α levels, while the adjuvant-only group showed both TNF-α and elevated IL-6. In contrast, the rLinKAP + Poly ICLC group displayed significantly increased TNF-α, IL-6, and IFN-γ levels, alongside low IL-10 and IL-4, indicating a Th1-biased immune response (Fig. 5D).

To assess infection control, we quantified parasite burden by qPCR. The untreated and adjuvant-only groups exhibited significantly higher parasite burdens compared with the rLinKAP-treated group, where parasites were detectable in only one animal (Fig. 5E). Notably, rLinKAP treatment achieved a 75% reduction in parasite burden in this experimental model. For comparison, miltefosine—the current gold standard for leishmaniasis treatment—demonstrated a 50% reduction in parasite load in a separate experiment with *L. infantum*-infected animals (Fig. S4). Within the experimental model evaluated, these findings suggest that rLinKAP may serve as a promising candidate for controlling parasitic infection.

## DISCUSSION

The development of prophylactic and therapeutic vaccines against visceral leishmaniasis (VL) has been pursued for decades, with various candidate vaccines currently undergoing testing. Still, none have become available for implementation in human vaccination (13, 41). Therefore, efforts toward antigen discovery and their incorporation into effective vaccine formulations are still needed.

In this study, we selected the LinKAP as a potential vaccine target for VL and further characterization. This protein was previously identified through immunoproteomics (10). Although other approaches have been used to discover new *Leishmania anti*gens as vaccine candidates (25, 26, 42), LinKAP has been identified by a reverse vaccinology strategy using sera of mice that were partially protected by vaccination with total parasite extracts, adjuvanted with Poly ICLC and Montanide, attempting to recognize the antigens associated with protective responses (10).

LinKAP is highly conserved among *Leishmania* species, a characteristic that may be explored for testing a single vaccine against different forms of leishmaniasis. LinKAP-related sequences were also identified in *Trypanosoma* and *Endotrypanum* genomes. The evolutionary divergence placing *Leishmania* and *Trypanosoma* in different branches of a phylogenetic tree indicates the presence of sequence differences that may influence LinKAP’s immunogenic properties for each parasite due to amino acid and epitope variations. On the other hand, the presence of conserved domains containing amino acid repeats in trypanosomatid genera that diverged approximately 100 million years ago (28, 33) suggests that LinKAP plays an essential role in the biology of these parasites and has been maintained under high selective pressures.

LinKAP has a distinctive amino acid repetitive structure, like other antigenic and immunogenic proteins in protozoa, which have been previously explored as vaccine targets and highlighted as potential virulence factors for *Leishmania spp*. and other trypanosomatids (43, 44). Some of these repetitive proteins, such as amastins, belong to families, which are highly expressed in amastigotes, indicating that *Leishmania* parasites actively exploit repetitive proteins for intracellular parasitism and immune evasion in mammalian hosts (45, 46). Another notable example is the amastigote-specific protein 2 (A2), member of a repetitive protein family found in *L. donovani* (47). The *A2* proteins are associated with differentiation, migration to visceral organs, and protective responses against temperature stress (32, 48). A2 proteins range from 42 to 100 kDa, depending on the number of repeats in each gene copy (49, 50). Although LinKAP is expressed from a single-copy gene, the size of the protein likely varies in its repetitive region among different *Leishmania* species, generating proteins with molecular weights ranging from 75 to 150 kDa.

Epitope mapping revealed the presence of CD4+ and CD8+ epitopes within these repetitive units, similar to what has been described for *A2* proteins (43). These findings correlate with evidence that *A2* is a promising antigen for VL vaccine formulations. Indeed, vaccine formulations containing *A2* epitopes have been successfully tested in experimental models, including rhesus monkeys (51–53, 54). Moreover, a prophylactic vaccine for canine VL (53, 55, 56) containing the recombinant *A2* protein was licensed and commercialized in Brazil for several years. This vaccine also demonstrated remarkable results as a therapeutic vaccine for treating hunting dogs with VL (42, 55, 57, 58).

Protein domains are valuable indicators for predicting function (59). In LinKAP, two domains were identified, providing insights into its role: the trichohyalin-plectin-homology (TPH) domain and a TolA protein domain. The TPH domain has been associated with mitochondrial movement, while TolA is part of the Tol–Pal complex, which plays a role in maintaining the integrity of the outer membrane (24, 59). *E. coli* TolA is an inner membrane protein with three distinct domains. In LinKAP, the TolA domain is predicted to be present and is located within the protein’s alpha-helical regions. A surface protein similar to TolA, known as TolT, has been characterized in the *T. cruzi* parasite and features an extended and unique alpha-helical structure. Although these domains may primarily serve a structural function, TolT has been shown to trigger specific T-cell immune and protective responses in mice immunized with this protein (60, 61).

LinKAP has been originally annotated in *Leishmania* genomes as a kinetoplastid-associated protein (KAP), referring to the specific mitochondrial region containing the kinetoplastid’s mitochondrial genome. Different KAPs have been studied in various protozoa, including *Leishmania*, *Trypanosoma*, and *Crithidia* (62–64). Subcellular fractionation and immunofluorescence experiments are consistent with LinKAP mitochondrial localization. After differential centrifugation of *Leishmania* subcellular structures, LinKAP was enriched in fractions containing other mitochondrial proteins. However, confocal microscopy revealed that LinKAP co-localizes with a mitochondrial marker and is distributed throughout the mitochondria rather than being confined to the kinetoplast region. So far, we have not identified LinKAP’s functions. However, given its distinctive structure, the high degree of sequence conservation, and the crucial roles of mitochondria in trypanosomatid biology, further studies are warranted to elucidate LinKAP’s function. These studies may also contribute to a better characterization of mitochondria in this important group of protozoan parasites.

Immunization with the recombinant truncated version of LinKAP (rLinKAP) and the Poly ICLC adjuvant induced a specific Th1 immune response in BALB/c mice and hamsters. Th1-type adjuvants are commonly selected for VL vaccine formulations because they stimulate antigen-presenting cells (APCs), thereby promoting T-cell activation and polarization of the adaptive immune response (65). Among the new generation of adjuvants, synthetic polyinosinic-polycytidylic acid (Poly IC), when combined with the stabilizers carboxymethylcellulose and polylysine (Poly ICLC), acts as a potent activator of Toll-like receptor 3 (TLR3) and MDA5 from the RIG-I family. These innate immune receptors recognize double-stranded RNA and promote T-cell-mediated immunity (66, 67). Notably, Poly ICLC has also been used in preclinical trials for COVID-19 (68), Chagas disease vaccines (69), and several cancer immunotherapy clinical trials (66, 70, 71). The fact that Poly ICLC has already been tested in clinical trials may accelerate the development of LinKAP-based vaccines for human use (70).

The immune response induced in vaccinated animals was characterized by IFN-γ production, low IL-10 levels, and a predominance of IgG2a antibodies. Additionally, hamsters vaccinated with rLinKAP exhibited increased antibody titers compared with the control groups. Although the humoral immune response may not directly correlate with protection, IgG1 and IgG2a are considered markers of T-cell activation (72). Studies have shown that, during *Leishmania* infection, an increase in IgG1 levels relative to IgG2a is associated with disease progression, whereas the opposite is linked to protective immunity (73). Immunized animals exhibited significantly reduced parasite burden in the liver and spleen compared with control groups. Altogether, these data suggested that rLinKAP is a promising candidate for composing a vaccine against leishmaniasis.

Based on previous evidence of the benefits of therapeutic protein-based vaccines for leishmaniasis, we also investigated the potential of our rLinKAP vaccine formulation to improve parasite control and immune responses in mice infected with *L. infantum*. Our findings indicate that vaccination with rLinKAP elicits the production of crucial cytokines involved in resistance to parasite infection, including IFN-γ, and protective immune responses against *Leishmania* infection. Indeed, other studies have explored immunotherapy approaches (74–77) that promote a more balanced Th1/Th2 immune response toward a Th1 profile, which may enhance parasite control. These interventions aim to control the levels of Th2 cytokines, such as IL-10 or IL-4, individually or in combination while increasing IL-12 and IFN-γ levels (75, 78, 79). Thus, a robust Th1 response provides a rational basis for effective therapeutic intervention (40), increasing host resistance to *Leishmania* infection.

Vaccines have long been considered promising immunotherapeutic interventions for leishmaniasis (80). Although it has been suggested that therapeutic vaccines will likely be used in combination with chemotherapy (41), our data indicate that treatment with the rLinKAP formulation, combined with Poly ICLC, led to a significant reduction in parasite load in a highly susceptible animal model. Nonetheless, this approach may be improved with established chemotherapeutic alternatives and protocols (81–83).

Overall, the results presented in this study identify LinKAP as a novel conserved protein of *Leishmania* spp., characterized by a repetitive amino acid structure and conserved mitochondrial-associated domains. Given these characteristics and the unique features of mitochondria in kinetoplastid protozoa, further studies are warranted to elucidate the function of LinKAP and its relevance in host–parasite interactions. Additionally, our findings demonstrate that rLinKAP contains T-cell epitopes and represents a promising candidate for both prophylactic and therapeutic vaccines against *Leishmania* infection.

## MATERIALS AND METHODS

### Evolutionary analysis of the LinKAP protein-encoding gene

The evolution of the LinKAP gene was investigated within trypanosomatid genera using the available genome and annotation data. Amino acid sequences were retrieved from the TritrypDB database (84), version 58, from nine different species of *Leishmania*—L. *amazonensis, L. arabica, L. braziliensis, L. donovani, L. gerbelli, L. infantum, L. major, L. mexicana, and L. panamensis—as* well as from *T*. *cruzi and T. brucei*. Genes from different strains for all mentioned species were also considered. The alignment of the retrieved sequences was performed using the MAFFT v7 program (85), and the resulting file was used as input to generate a maximum likelihood (ML) phylogenetic reconstruction executed by the PhyML v3.0 software (86). The best substitution model was determined to be LG + I, based on the agreement between the Akaike Information Criterion (AIC) and the Bayesian Information Criterion (BIC) provided by the ProtTest 3.4.2 tool (87). Gene clusters were determined according to the genus to which the gene belongs. The LinKAP gene was also used to conduct an evolutionary analysis of the *Leishmania* genus, considering only sequences from this group. Seven amino acid sequences belonging to the same species (from different strains) were concatenated into a single sequence, and the phylogenetic tree was generated at the species level of *Leishmania*, following the previously described steps. The sequence of *Endotrypanum monterogeii* was used as an outgroup to root the tree.

### Rational design of the LinKAP recombinant protein (rLinKAP)

The LinKAP protein was sequenced for epitope prediction using the Immune Epitope Database (IEDB) for class I and NetMHCII for class II (https://www.iedb.org/) and BIMAS (https://bio.tools/hla_bind). Potential epitopes with affinity for mouse HLA-ABC, HLA-DR, and MHC-I/II were identified with a percentile rank threshold of ≤5. The selected region included epitopes with ≥80% identity across *L. infantum*, *L. donovani*, *L. amazonensis*, *L. brasiliensis*, *L. major*, and *L. mexicana*. Physicochemical properties, antigenicity, and solubility of the selected region were assessed using ProtParam (https://web.expasy.org/protparam/), VaxiJen v2.0 (https://www.ddg-pharmfac.net/vaxijen/VaxiJen/VaxiJen.html), and Protein-Sol (https://protein-sol.manchester.ac.uk/).

### Interferon gamma ELISpot assay

Female BALB/c mice (*n* = 6) were allocated into two groups: (i) a non-immunized control and (ii) immunized with recombinant rLinKAP + Poly ICLC. Thirty days after the final immunization, the animals were euthanized, and splenocytes were isolated for IFN-γ ELISpot assays. Ninety-six-well plates (MAHAS 4510, Millipore) were coated with 5 µg/mL anti-mouse IFN-γ capture antibody (MABTECH) in carbonate/bicarbonate buffer (pH 9.6) and incubated overnight at 4°C. The following day, plates were blocked for 1 h at 37°C (5% CO_2_) in RPMI 1640 supplemented with 5% FBS. A total of 1 × 10^6^ splenocytes in RPMI-10% FBS, rIL-2, and anti-CD28 (2 µg/mL) were seeded per well. Negative controls consisted of medium plus cytokines (rIL-2 and anti-CD28), while positive controls additionally included anti-CD3 (10 µg/mL) or concanavalin A (5 µg/mL). For specific stimulation, wells received a pool of rLinKAP-derived synthetic peptides (10 µg/mL). After 24 h at 37°C (5% CO_₂_), supernatants were discarded, and wells were washed four times with 0.5% PBS-Tween, followed by four washes with PBS. Plates were then incubated for 2 h at room temperature with biotinylated anti-IFN-γ (2 µg/mL), washed, and exposed to streptavidin–peroxidase (1:2,000) for 1 h. Spots were developed with DAB substrate in 0.1 M Tris-HCl (pH 7.5) plus H_₂_O_2_, washed with water, and air-dried overnight. Spot-forming units were counted on an automated reader (Mabtech IRIS). Results are presented as Δ (delta), calculated by subtracting the number of spots in RPMI-only wells from those in peptide-stimulated wells.

### Expression and purification of LinKAP recombinant protein

The DNA encoding the rLinKAP protein was commercially synthesized and cloned into the pET-24a expression vector (GenScript) to transform *E. coli* BL21(DE3) cells. Positive clones were cultured in LB medium, and protein expression was induced with 0.5 mM IPTG at 37°C. The His-tagged LinKAP protein was purified by affinity chromatography using a HisTrap HP column (GE Healthcare) per the manufacturer’s instructions. Endotoxins were removed using the ToxinEraser Endotoxin Removal Kit (GenScript).

### Circular dichroism spectroscopy

The secondary structure preferences of rLinKAP were investigated by Circular Dichroism (CD) for the protein diluted 30 times in water (final concentration of 0.1 mg/mL). The rLinKAP buffer was diluted in the same proportion and used as a blank in the experiment. The profile of the secondary structure of the proteins was evaluated by CD using a Jasco J-1500 spectropolarimeter (Jasco Corporation, Tokyo, Japan) equipped with a Koolance temperature control system (Koolance, USA). The dichroic spectra were obtained in a quartz cuvette with an optical path length of 0.1 cm at 25°C. Ten dichroic spectra were accumulated for each sample at far-ultraviolet wavelengths (FAR-UV 190–260 nm) with 0.1 nm intervals, a scan rate of 50 nm/min, a response time of 2 s, and a bandwidth of 1.7 nm. The mean readings were corrected by subtracting buffer contributions.

The ellipticity obtained for each wavelength (θλ) was converted into molar ellipticity by residues [θ]MRW using the following equation:


$$[\theta {]}_{MRW}=\theta /(10\times cr\times l),$$

where cr is the molar concentration per amino acid residue, and *l* is the optical path length of the quartz cuvette (cm). The value of [θ]_MRW_ is expressed in degrees.cm^2^/dmol. The secondary structure content of the protein was estimated on the Bestsel web server (http://bestsel.elte.hu/index.php).

### Production of polyclonal antibodies against rLinKAP

A male New Zealand White rabbit was immunized every 14 days with three doses of 100 µg of purified rLinKAP protein emulsified with aluminum hydroxide adjuvant (30%, vol/vol). Serum samples were collected after each immunization to assess the antibody response to rLinKAP by ELISA. All procedures adhered to ethical guidelines for the use of laboratory animals. IgG antibodies were purified using an affinity chromatography column containing protein A resin (HiTrap Protein A, GE) on an ÄKTAprime system (GE) and used in the Western blot analysis.

### MALDI-MS measurement

MALDI-MS measurements were performed in positive linear mode using MALDI-8020 (Shimadzu Corporation, Japan) equipped with a 200 Hz solid-state laser, 355 nm. The samples were co-crystallized with sinapic acid (SA) matrix (1:1, vol/vol) on FlexiMass-SR48 (Shimadzu Corporation, Japan). The instrument was mass-calibrated using two peaks with *m*/*z* 33,215.55 and 66,431.077 from bovine serum albumin. Three mass spectra were acquired in the range of *m*/*z* 8,000–50,000 with ion gate blanking 9,000. The results were analyzed on MALDI Solutions Data Acquisition Shimadzu Corporation, Japan.

### Cultivation of parasites and total extract

*Leishmania* promastigotes were cultured in Schneider’s medium supplemented with 1% penicillin-streptomycin 100× (Gibco) and 10% fetal bovine serum (FBS) at 26°C in a biochemical oxygen demand (BOD) incubator, with subculturing every 5 days. For total extract preparation, 1 × 10⁷ parasites were centrifuged at 1,450 × *g* for 10 min, washed twice with phosphate-buffered saline (PBS), and lysed by sonication. Protein concentration was determined using the Bradford method.

Protein expression analysis was performed on extracts from *L. infantum*, *L. amazonensis*, *L. braziliensis*, *L. mexicana*, and *L. donovani*. Samples (30 µg) were separated by polyacrylamide gel electrophoresis and transferred to nitrocellulose membranes. Membranes were incubated with rabbit-derived anti-LinKAP polyclonal antibodies, diluted 1:1,000 in TBS-T, for 1 h at room temperature with agitation. Pre-immune serum was used as a negative control. Membranes were then incubated with peroxidase-conjugated anti-rabbit IgG (Sigma-Aldrich), diluted 1:10,000 in TBS-T, for 1 h at room temperature.

### Live cell assays with endogenous labeling of the LinKAP protein

The gene encoding LinKAP was tagged in *L. mexicana* T7/Cas9 parental cells, which express T7 RNA polymerase and Cas9 endonuclease (88). Primers for sgRNA generation and repair templates were designed with LeishGEdit (http://leishgedit.net/) (89) and included binding sites for pTBlast_v1 or pTPuro_v1 plasmids, as well as 30-nt homology arms for homologous recombination.

PCR amplification of repair cassettes was conducted with 30 ng of plasmid (pTBlast_v1 or pTPuro_v1), 0.2 mM dNTPs, 2 µM primers, 1 U Q5 DNA polymerase (NEB), in a final volume of 40 µL. The PCR program included an initial denaturation at 94°C for 5 min, followed by 45 cycles of 94°C for 30 s, 65°C for 30 s, and 72°C for 2 min 15 s, with a final extension at 72°C for 7 min. sgRNAs were amplified similarly in 20 µL reactions.

For transfection, *L. mexicana* T7/Cas9 promastigotes (10⁶ cells) were centrifuged, washed in PBS, and resuspended in P3 solution (Lonza). Purified PCR products were transfected using the Amaxa 4D-Nucleofector (Lonza) with program FI-115, followed by recovery in HOMEM medium with 20% FBS and 1% penicillin/streptomycin. Negative controls were transfected with water instead of DNA. After 16 h at 25°C, cells were selected in HOMEM medium with 150 µg/mL blasticidin and 75 µg/mL puromycin. Genomic DNA was extracted using the Qiagen DNeasy Blood and Tissue Kit, and diagnostic PCR was performed with Q5 DNA Polymerase (NEB) as per the manufacturer’s instructions.

### Isolation of organelles from *Leishmania*

Cells were lysed by nitrogen cavitation to isolate organelles from *Leishmania*, as described previously (90), with some modifications. *L. mexicana* promastigotes (3 × 10⁹) were resuspended in 10 mL of sucrose buffer (0.25 M sucrose, 10 mM HEPES-KOH [pH 7.4], 1 mM EDTA, protease inhibitors) and subjected to nitrogen cavitation on ice at 1,000 psi for 15 min. The lysate was centrifuged at 200 × *g* for 5 min to remove unlysed cells, and the supernatant was then fractionated by differential centrifugation (1,000 × *g* to 120,000 × *g*, 4°C). Fractions were analyzed by Western blotting. For LinKAP detection, the membrane was incubated with polyclonal anti-rLinKAP antibodies (rabbit) and antibodies against compartment markers: (i) nucleus, anti-Myc; (ii) glycosome, anti-mevalonate kinase; (iii) mitochondria, anti-HMGCR; (iv) ER, anti-BiP; (v) cytoskeleton, anti-β-tubulin; and (vi) cytosol, oligopeptidase B and elongation factor 1-α. Densitometric analyses were performed using ImageJ (NIH). For each protein, the densitometric data were normalized in relation to the fraction with the strongest signal (100%), excluding the whole cell lysate (WCL).

### Cellular localization assay of LinKAP by confocal microscopy in promastigote forms of *Leishmania* species

Promastigotes of *L. infantum* and *L. amazonensis* were centrifuged at 1,450 × *g* for 10 min at 4°C, washed with PBS, and fixed in 2% paraformaldehyde in PBS. Cells were permeabilized with 0.1% PBS-T for 10 min at room temperature, followed by treatment with 1M glycine for 10 min. Parasites were adhered to 0.1% poly-L-lysine-coated slides and blocked with 1% BSA in PBS at room temperature.

The rLinKAP protein was detected with a primary anti-LinKAP antibody (1:1.000) generated in rabbits incubated for one hour at 4°C. Negative controls included pre-immune serum or omission of the primary antibody. After washing with PBS-T, Alexa Fluor 488-conjugated anti-rabbit IgG (Invitrogen, 1:1000) was applied for 1 h at room temperature. Nuclei and kinetoplasts were stained with 1 µg/mL DAPI (Life Technologies) for 5 min at room temperature. Slides were washed in PBS-T, mounted with ProLong Gold reagent (Life Technologies), and covered with glass coverslips.

### Cellular localization assay of LinKAP by fluorescence microscopy in amastigote forms of *Leishmania*

Bone marrow-derived macrophages (BMDMs) were differentiated from committed myeloid progenitors isolated from BALB/c mice and infected with promastigote *L. mexicana* at a 10:1 parasite-to-macrophage ratio. Macrophages (4 × 10⁴ cells/well) were plated in complete medium (DMEM, 2% house serum, L-glutamine, penicillin/streptomycin) and incubated with parasites for 48 h at 37°C with 5% CO_₂_. Following infection, cells were washed with PBS, permeabilized, and blocked with 1% BSA in PBS at room temperature.

The rLinKAP protein was labeled by incubating with a primary rabbit anti-LinKAP antibody (1:1,000) for 1 h at 4°C. Negative controls included pre-immune serum or omission of the primary antibody. After PBS-T washes, Alexa Fluor 488-conjugated anti-rabbit IgG (Invitrogen, 1:1,000) was applied for 1 h at room temperature. Nuclei and kinetoplasts were stained with 1 µg/mL DAPI (Life Technologies) for 5 min. Slides were washed in PBS-T, mounted with ProLong Gold (Life Technologies), and covered with coverslips.

### Immunization protocols and challenges

BALB/c mice (*n* = 10) and hamsters (*n* = 4) were immunized subcutaneously with three doses of vaccine formulations at 21-day intervals. The recombinant protein group received 10 µg LinKAP and 50 µg Poly ICLC adjuvant in a total volume of 100 µL per dose. Control groups received either sterile saline or 50 µg Poly ICLC alone. Thirty days after the final dose, mice were euthanized, and serum and spleen samples were collected to assess B and T cell responses. The remaining animals were challenged with *L. infantum* promastigotes (MHOM/BR/74/PP75) (10⁷ parasites for mice, 10⁸ for hamsters), and parasite load was evaluated 30 days post-infection. All samples were analyzed in duplicate to ensure reproducibility.

### Infection and therapeutic protocol

BALB/c mice (*n* = 4 per group) were infected with 10⁷ *L. infantum* promastigotes (MHOM/BR/74/PP75). Sixty days post-infection, animals received the same formulations as in the prophylactic protocol for 1 week. Fifteen days after the final dose, animals were euthanized, and serum and spleen samples were collected to assess B- and T-cell responses. Parasite load in the liver and spleen was quantified by qPCR. All samples were analyzed in duplicate to ensure reproducibility. To the treatment of BALB/c mice with miltefosine, initially, BALB/c mice infected with *Leishmania infantum* (MHOM/BR/74/PP75) were treated with miltefosine (10 mg/kg dose, orally). Milteforan 2%, Virbac, France) twice a week, starting 2 weeks post-infection. After 7 weeks of infection, the animals were euthanized, and the spleen and liver were collected and processed for parasite burden quantification using the limiting dilution assay.

### Immunological assays

To evaluate IFN-γ and IL-10 production, 1 × 10⁶ splenocytes from mice were incubated at 37°C with 5% CO_₂_ for 48 h with four stimuli: (i) 10 µg/mL rLinKAP, (ii) 10 µg/mL RPMI (unstimulated control), and (iii) concanavalin A (positive control). IL-10 and IFN-γ levels in supernatants were quantified using Duoset ELISA kits (R&D Systems) per the manufacturer’s instructions. Additional cytokine levels were measured using the cytometric bead array (CBA) Mouse Th1/Th2/Th17 Cytokine Kit (BD), following the manufacturer’s protocol.

Total IgG, IgG1, and IgG2a levels were determined by ELISA. Briefly, 96-well plates were coated with rLinKAP protein (100 ng/100 µL/well) for 12 h. Mouse serum was added at a 1:500 dilution and incubated for 1 h at 37°C. Mouse anti-IgG antibodies (total IgG, IgG1, and IgG2a) conjugated with peroxidase (Southern Biotech) were added at a 1:40,000 dilution and incubated for 1 h at 37°C. TMB substrate (100 µL, 2.08 mM; MOSS) was added, and the reaction was stopped with 100 µL of 0.5M sulfuric acid. Absorbance was read at 450 nm using a Multiskan-Go microplate reader.

### Estimation of parasite load

To assess *L. infantum* infection, animals were euthanized, and the liver and spleen were harvested. For the prophylactic vaccine, viable parasites were quantified per milligram of tissue using the limiting dilution assay (LDA), while for the therapeutic vaccine, parasite load was measured by qPCR. Liver and spleen samples were homogenized in 3 mL of Schneider’s medium. After centrifugation at 50 *× g* for 1 min at 4°C to remove debris, the supernatant was centrifuged at 1,540 *× g* for 10 min at 4°C. The pellet was resuspended in 500 µL of Schneider’s medium and serially diluted (1:10) in 96-well plates (200 µL final volume). Plates were incubated at 26°C for 14 days, with weekly evaluation of parasite presence.

For qPCR, DNA was extracted and diluted to 100 ng/µL in nuclease-free water. SYBR Green qPCR was performed using primers specific for *L. infantum* KDNA (EU437407) (KDNA fw: 5′-CCTATTTTACACCAACCCCCAGT-3′, KDNA rv: 5′-GGGTAGGGGCGTTCTGCGAAA-3′) and *Mus musculus* β-actin (NM_007393.5) (Actin fw: 5′-CTCCATGAAGTGTGACGTT-3′, Actin rv: 5′-ATCTCCTTCTGCATCCTGTCAG-3′). Reactions included a final volume of 15 µL, with each primer at 10 nM concentration and 100 ng of DNA template. Standard curves were generated with eight serial dilutions (0.1–100,000 pg) of target DNA. All samples and negative controls were run in duplicate, and parasite load was estimated as the number of *Leishmania* cells per 10⁶ mouse cells.

### Animals

Female BALB/c mice (6 to 8 weeks old) were obtained from the Institute of Biological Sciences at the Federal University of Minas Gerais (Belo Horizonte, Brazil) and used for vaccine testing. New Zealand White rabbits were also used to produce polyclonal antibodies specific for the recombinant protein rLinKAP.

### Statistical analysis

Statistical analysis was performed using GraphPad Prism 8.4.6. Outliers were first detected using the Grubbs‘ test, followed by the Shapiro–Wilk test to assess the normality of the data. The tests used in each data analysis are detailed in the supplemental material. Generally, comparisons between groups were carried out using the unpaired *t*-test with Welch’s correction, assuming a Gaussian distribution of the data without assuming equal variances between groups, the Mann–Whitney *U* test, Kruskal-Wallis test, or ANOVA, depending on the data distribution. Statistical differences were considered significant when *P*-values were ≤0.05. Significance levels were denoted as follows: *P ≤ 0.05, **P ≤ 0.01, and ***P ≤ 0.001.

## Data Availability

The authors declare that all data supporting the findings of this study are available within the paper and its supplemental information files. Additional data related to this study are available from the corresponding author upon reasonable request.

## SUPPLEMENTAL MATERIAL

Supplemental Material msphere.00097-25-s0001.pdf Supplemental figures and table.

ASM does not own the copyrights to Supplemental Material that may be linked to, or accessed through, an article. The authors have granted ASM a non-exclusive, world-wide license to publish the Supplemental Material files. Please contact the corresponding author directly for reuse.
