# Supplementary material for: Characterization of a novel Leishmania antigen containing a repetitive domain and its potential use as a prophylactic and therapeutic vaccine
Source: mSphere. 2025 Apr 22;10(5):e00097-25. doi: 10.1128/msphere.00097-25 (PMC12108087; doi:10.1128/msphere.00097-25)
Supplement: Supplemental Material — Supplemental figures and table. [file msphere.00097-25-s0001.pdf]

SUPPLEMENTAL MATERIAL

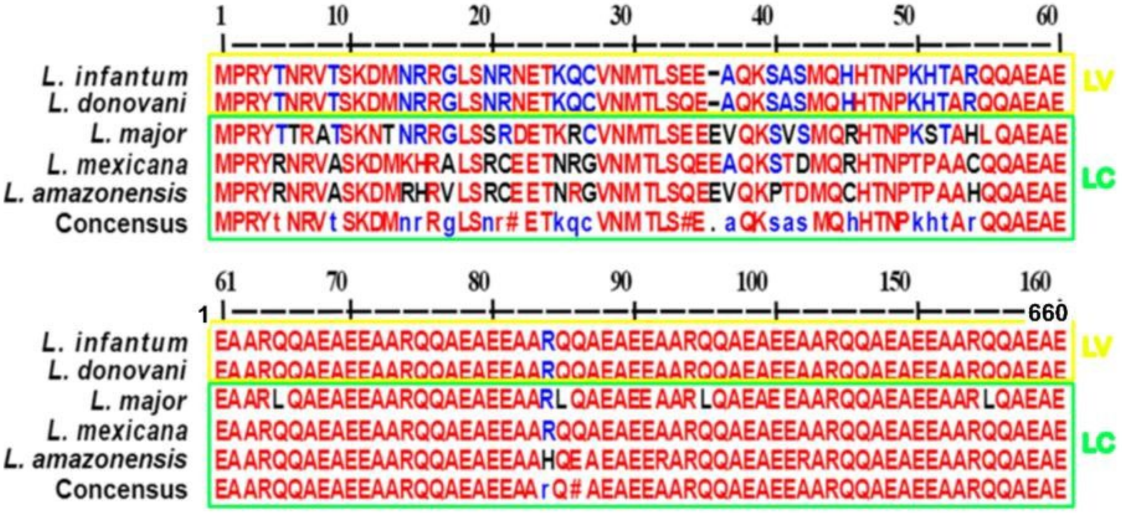

Supplementary Fig. 1. Sequence alignment among *Leishmania* species. The alignment was performed using tblastx. Only the largest region with a similarity greater than 85% is shown, which corresponds to the repetitive region of the protein. The yellow rectangle highlights the species related to visceral leishmaniasis, and the green rectangle the species related to cutaneous leishmaniasis.

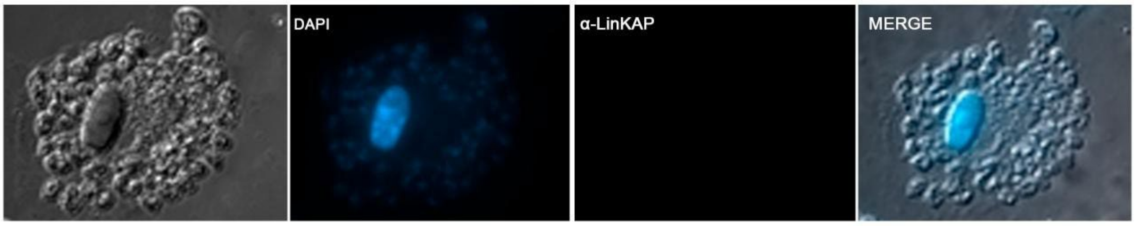

Supplementary Fig. 2. *Negative control for detecting LinKAP in amastigote forms of Leishmania.* Immunofluorescence microscopy images demonstrate LinKAP expression in amastigote cells. DAPI was used to stain the nucleus (second panel), while the anti-LinKAP antibody was applied (third panel)

without a secondary antibody to verify the staining specificity. The absence of signal in the α-LinKAP channel confirms the specificity of the antibody for LinKAP detection, as observed in Figure 1F. Merged images (right panel) show DAPI localization without non-specific LinKAP staining, validating the LinKAP detection in the amastigote stage and excluding the possibility of false-positive results due to antibody cross-reactivity or background fluorescence.

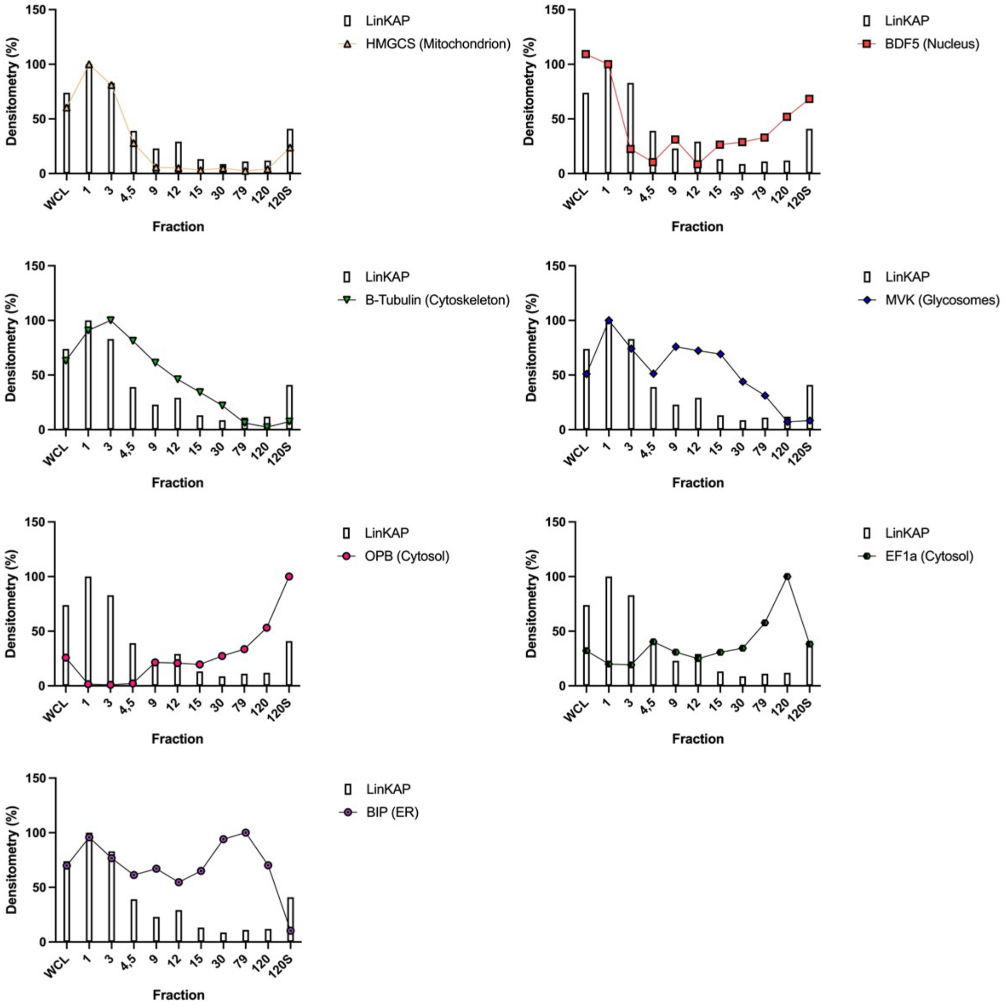

Supplementary Fig. 3. Densitometric analysis of LinKAP distribution across subcellular fractions obtained by differential centrifugation. Each graph

represents the densitometric distribution of LinKAP compared to specific subcellular markers, including β-tubulin (cytoskeleton), OPB and EF1α (cytosol), HMGCS (mitochondria), MVK (glycosomes), BIP (endoplasmic reticulum), and BDF5 (nucleus). Although LinKAP was detected across multiple fractions, it showed enrichment in low-speed pellet fractions, displaying a similar distribution pattern to the mitochondrial marker HMGCS. Densitometric analysis was performed using ImageJ (NIH) and normalized to the fraction with the highest signal (100%), excluding the whole cell lysate (WCL).

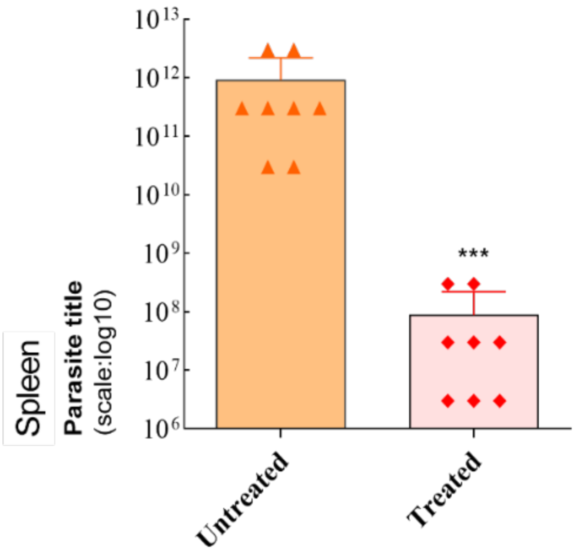

Supplementary Fig. 4. Reduction of spleen parasite load in *Leishmania infantum*-infected mice after miltefosine treatment. Infected mice were divided into two groups: Untreated (no treatment) and Treated (treated with miltefosine). Parasite load in the spleen was quantified and expressed on a logarithmic scale (log10). The treated group significantly reduced parasite burden compared to the untreated group. Statistical significance is represented as *p <

0.05, **$p < 0.01$, and *$p < 0.001$ (Mann-Whitney U test). Dots represent individual values for each animal.

Statistical Analysis Methods for Figures 2E, 4, and 5

Fig. 2E: Statistical analysis was conducted using an unpaired Student's t-test.

Fig. 4: Statistical analysis for panels B-F was conducted using one-way ANOVA followed by Tukey’s multiple comparison test. The Mann-Whitney test was used for panels G and H to compare differences between the vaccinated group with Poly ICLC and the vaccinated group with saline. The Student's t-test was used for panels I and J.

Fig. 5: Statistical analysis for panel B was conducted using the Kruskal-Wallis test for IgG total and IgG1, and ANOVA for IgG2a due to normality assumptions. The Mann-Whitney test was used for panel C. Panel E was analyzed using ANOVA.

Supplementary Table 1: The top ten list of ranking peaks for each replicate analyzed of LinKAP on MALDI-MS:

Mass list of ten more intensities, replicate one.

Mass (Da)	Intensity (mV)	Intensity (%)	Area (mV)	Area (%)	Resolution	S / N
23013.486	1.49	100	672.58	100	33	20
23228.406	1.28	86	404.51	60	29	17

23484.449	0.87	59	295.28	44	23	12
46049.211	0.62	41	460.74	69	32	17
46502.404	0.58	39	381.93	57	31	16
47000.324	0.36	24	154.95	23	22	9
15362.459	0.32	22	93.97	14	19	3
15504.897	0.29	20	72.58	11	17	2
15044.438	0.23	15	66.84	10	12	2
15639.676	0.23	15	35.58	5	13	2

Mass list of ten more intensity, replicate two.

**Mass (Da)**	**Intensity (mV)**	**Intensity (%)**	**Area (mV)**	**Area (%)**	**Resolution**	**S / N**
23009.968	1.8	100	738.79	100	36	17
23205.801	1.59	88	642.71	87	32	15
23468.270	1.02	57	295.57	40	23	9
46064.776	0.7	39	499.84	68	35	14
23649.822	0.66	37	68.87	9	18	6
46479.046	0.66	36	427.7	58	34	13
15362.335	0.35	20	93.51	13	17	2
47007.019	0.35	19	77.17	10	22	6
15503.902	0.32	17	78.62	11	15	2
22457.293	0.28	15	19.82	3	8	2

Mass list of ten more intensity, replicate three.

Mass (Da)	Intensity (mV)	Intensity (%)	Area (mV)	Area (%)	Resolution	S / N
23027.899	13.84	100	7218.74	100	27	8
23228.479	12.37	89	4412.49	61	24	7
23500.487	8.92	64	2914.65	40	18	5
46054.891	6.92	50	5742.84	80	26	7
46509.669	6.51	47	4145.46	57	25	7
15371.544	4.42	32	1256.47	17	12	2
47028.167	4.3	31	1490.21	21	16	4
15516.317	4.11	30	1111.16	15	8	2
15670.545	3.45	25	656.95	9	3	1
15036.562	3.3	24	791.92	11	2	1
